# Comparison of the safety and efficacy of triple sequential therapy and transscleral cyclophotocoagulation for neovascular glaucoma in the angle-closure stage

**DOI:** 10.1038/s41598-018-25394-9

**Published:** 2018-05-04

**Authors:** Ying Hong, Yuntao Hu, Hongliang Dou, Changguan Wang, Chun Zhang, Zhizhong Ma

**Affiliations:** 1 Department of Ophthalmology, Peking University Third Hospital Key Laboratory of Vision Loss and Restoration, No. 49 North Garden Road, Haidian District, Beijing China; 2 Department of Ophthalmology, Beijing Tsinghua Chang Gung Hospital, No. 168 Litang Road, Changping District, Beijing China; 3 Department of Ophthalmology, Peking University International Hospital, No. 1 Life Garden Road, Changping District, Beijing China

## Abstract

To compare the efficacy and safety of triple therapy combining intravitreal injection of anti-vascular endothelial growth factor, trabeculectomy, and pan-retinal photocoagulation via binocular indirect ophthalmoscopy, with that of transscleral cyclophotocoagulation (TCP) to treat neovascular glaucoma in the angle-closure stage. Eighteen triple therapy patients and 25 TCP patients between May 2014 and May 2016 were retrospectively analysed. Anterior chamber puncture and anti-VEGF intravitreal injection were performed on the first day of sequential therapy. Trabeculectomy was performed 3–5 d after injection; pan-retinal laser photocoagulation via binocular indirect ophthalmoscopy was initiated 5–7 d later. The IOP of the triple therapy group was lower than that of the TCP group (15.2 ± 2.2 vs. 20.0 ± 8.5 mmHg) and fewer anti-glaucoma drugs were used (0.5 ± 1.0 vs. 0.6 ± 1.0) after treatment. The success rates of the two groups were 89% and 60% respectively (P = 0.032). The visual function of 94% of triple therapy patients was preserved or improved compared to 64% of TCP patients with statistical significance (P = 0.028). No patient in the triple therapy group showed hypotony or eyeball atrophy. Compared to TCP, triple therapy shows higher success rate, fewer complications, and attributes to visual function preservation.

## Introduction

Neovascular glaucoma (NVG) is a type of refractory glaucoma, which is usually secondary to retinal ischemia. The vascular endothelial growth factor (VEGF) released after retinal ischemia will result in neovascularization of the iris and anterior chamber angle. Fibrous vascular contraction in the angles and peripheral anterior synechiae will cause increased intraocular pressure (IOP) or even loss of vision^1–5^. Although the angle closure stage of neovascular glaucoma is the terminal stage of the disease, a considerable number of patients retain some visual function at this stage.

Traditional anti-glaucoma medications usually have minimal effect on the reduction of IOP in NVG. Filtering surgery in NVG patients is less successful because of intraoperative bleeding and filtering blebs neovascularization induced by VEGF. Although transscleral cyclophotocoagulation (TCP) may control IOP, it does not treat the primary retinal disease^6–10^. An injection of anti-vascular endothelial growth factor (anti-VEGF) has been reported to alleviate the symptoms^1–5^ and result in short-term control of intraocular pressure, which may enable additional measures to be employed for IOP control and treatment of retinal ischaemia^1^.

Therefore, we explored the efficacy of other treatments for NVG. In particular, we devised a combination therapy, known as triple sequential therapy, comprising intravitreal injection of anti-VEGF and trabeculectomy to control IOP, and pan-retinal photocoagulation (PRP) under indirect ophthalmoscopy. Subsequently, we compared the efficacy and complications between the triple sequential therapy and TCP for the treatment of NVG at the angle-closure stage.

## Results

### Intraocular pressure measurement

In the triple sequential therapy group, the IOP of patients before treatment was 43.5 ± 5.1 (36–52) mmHg, with 5.1 ± 0.5 (4–6) types of anti-glaucoma drugs. At the last follow-up after treatment, the IOP had decreased to 15.2 ± 2.2 (12–19) mmHg, with0.5 ± 1.0 (0–3) types of anti-glaucoma drugs.

In the TCP group, the IOP was 41.8 ± 9.0 (30–68) mmHg before treatment with 5.0 ± 0.9 (4–6) types of anti-glaucoma drugs. After treatment, the IOP decreased to 20.0 ± 8.5 (2–30) mmHg, and the number of drug types used decreased to 0.6 ± 1.0 (0–3).

The pre-treatment IOP and number of anti-glaucoma drugs used were not significantly different between the triple sequential therapy and TCP groups (t = −0.651, P = 0.519 and t = −0.292, P = 0.772, respectively). The post-treatment IOP and number of anti-glaucoma drugs used were slightly lower in the triple sequential therapy group than in the TCP group, although the difference was not significant (t = 1.984, P = 0.056 and t = 0.050, P = 0.961, respectively; Table 1).

**Table 1:** The results and demographic characteristics of the triple sequential therapy group and TCP groups.

	Triple Group	TCP Group	t/χ^2^	P
Patients	18	25	—	—
Gender (F/M)	15/3	19/6	0.340	0.712
Age (years) (Range)	61.9 ± 13.9 (38–84)	65.0 ± 11.3 (45–80)	2.467	0.124
IOP_pre_ (mmHg) (Range)	43.5 ± 5.1 (36–52)	41.8 ± 9.0 (30–68)	0.651	0.519
Anti-glaucoma Eye Drops (Range)	5.1 ± 0.5 (4–6)	5.0 ± 0.9 (4–6)	−0.292	0.772
IOP_post_(mmHg)	15.2 ± 2.2 (12–19)	20.0 ± 8.5 (2–30)	1.984	0.056
Anti-glaucoma Eye Drops (Range)	0.5 ± 1.0 (0–3)	0.6 ± 1.0 (0–3)	0.050	0.961
BCVA				
Improved	4	2	10.464	0.001
Maintained	13	14		
Decreased	1	9		
Retinal Disease				
CRAO	1			
CRVO	6	6		
BRVO	5	5		
CRAO & CRVO	2			
DR	4	9		
Undetermined		5		

BCVA, best corrected vision acuity; BRVO, branch retinal vein occlusion; CRAO, central retinal artery occlusion; CRVO, central retinal vein occlusion; DR, diabetic retinopathy; IOP, intraocular pressure; TCP, transscleral cyclocoagulation.

### Best corrected visual acuity (BCVA)

The BCVA of all patients was lower than counting fingers before treatment. The BCVA in the triple sequential therapy group ranged from light perception (LP)/1 m to counting fingers (CF)/20 cm. After treatment, the BCVA was found to have improved in four cases, was at the pre-treatment level in 13 cases, and decreased in one case (Table 1). The BCVA in the TCP group ranged from LP/1 m to CF/20 cm before surgery. After surgery, the BCVA was found to have improved in two cases, was at the preoperative level in 14 cases, and had decreased in nine cases (Table 1). In the triple sequential therapy group, the visual function of 94% (17/18) of the patients was preserved or improved after treatment. The corresponding ratio was 64% (16/25) in the TCP group, with a significant difference between the two groups (χ^2^ = 6.247; P = 0.028).

### Neovascular regression

In all cases, observation of the anterior chamber structures was difficult before treatment, a large number of new blood vessels were present on the surface of the iris, and the pupils were rigid.

**Figure 1:**
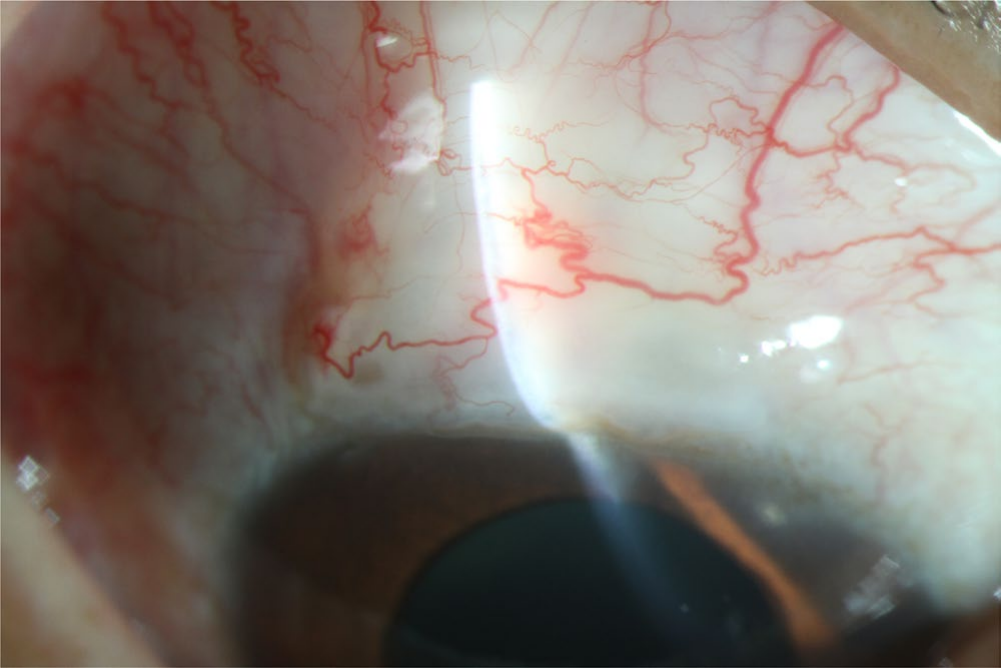
The Slit lamp photo of the filtering bleb after trabeculectomy in patients with neovascular glaucoma. The filtering bleb is present, the cornea is clear, and the neovascularisation of the iris is regressed.

In the triple sequential therapy group, complete regression of neovascularisation on the surface of the iris was observed in 16 eyes (89%). Among those, the neovascular regression occurred after anti-VEGF treatment in eight eyes and after trabeculectomy in six eyes. In the remaining two eyes, neovascular regression occurred after the completion of PRP. Anterior segment imaging after trabeculectomy revealed the presence of filtering blebs, a clear cornea, and regression of the neovascularisation of the iris (Fig. 1). In the TCP group, complete neovascular regression occurred in 10 eyes (40%; χ^2^ = 10.464; P = 0.001).

### Fundus diseases

**Figure 2:**
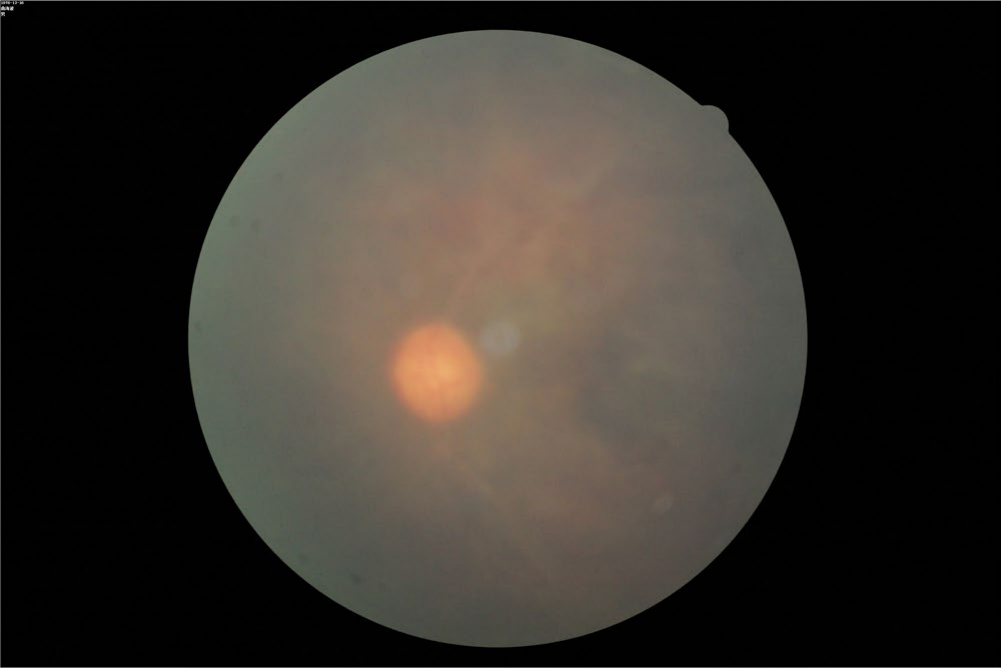
The fundus is very blurry before triple sequential therapy. The figure depicts that viewing of the retinal condition was difficult before treatment due to corneal oedema.

All patients presented with corneal oedema, and observation of the retina was difficult before treatment (Fig. 2).

**Figure 3:**
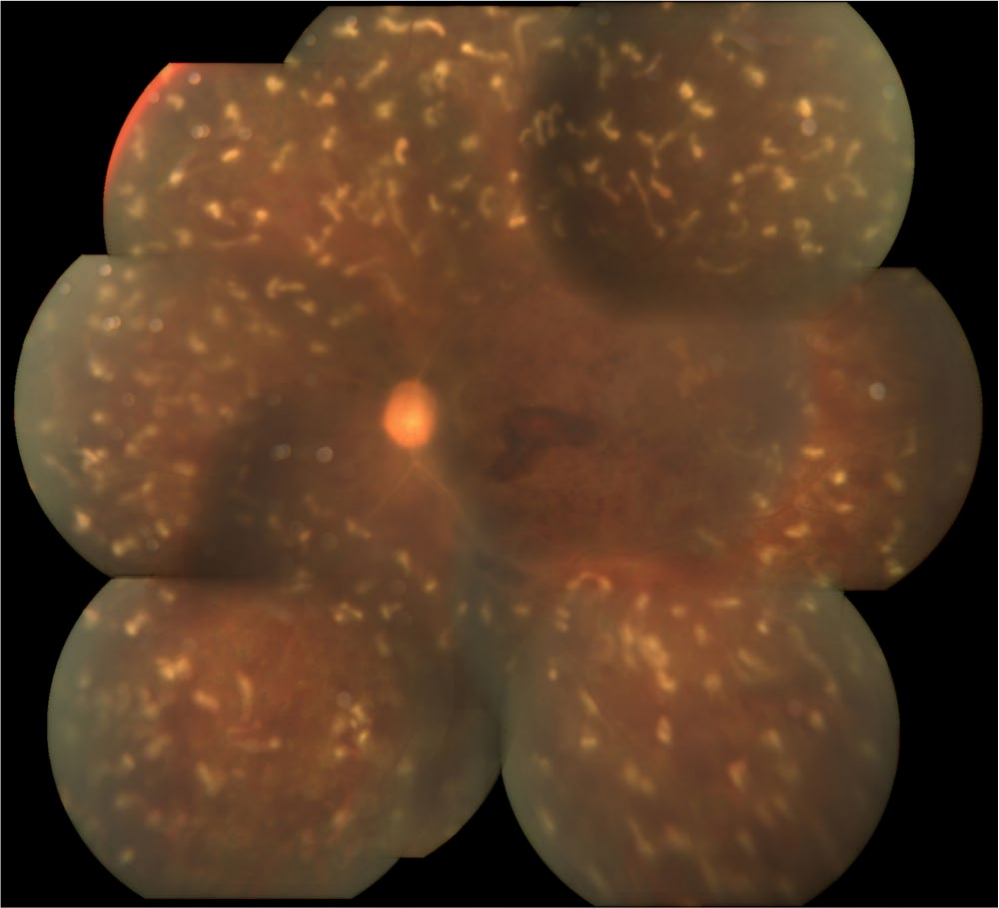
The fundus of the same patient after triple sequential therapy. Corneal oedema regressed after treatment. The retinal condition and laser spot could be seen after treatment.

Corneal oedema regressed after treatment. In the triple sequential therapy group, one eye was diagnosed with central retinal artery occlusion (CRAO), six with central retinal vein occlusion (CRVO), five with branch retinal vein occlusion (BRVO), four with diabetic retinopathy (DR; Fig. 3), and two with combined retinal artery and vein occlusion. In the TCP group, CRVO was confirmed in six eyes, BRVO in five eyes, and DR in nine eyes; the diagnosis of the remaining five eyes was not clear due to inability to view the fundus.

### The success rate and complications

**Figure 4:**
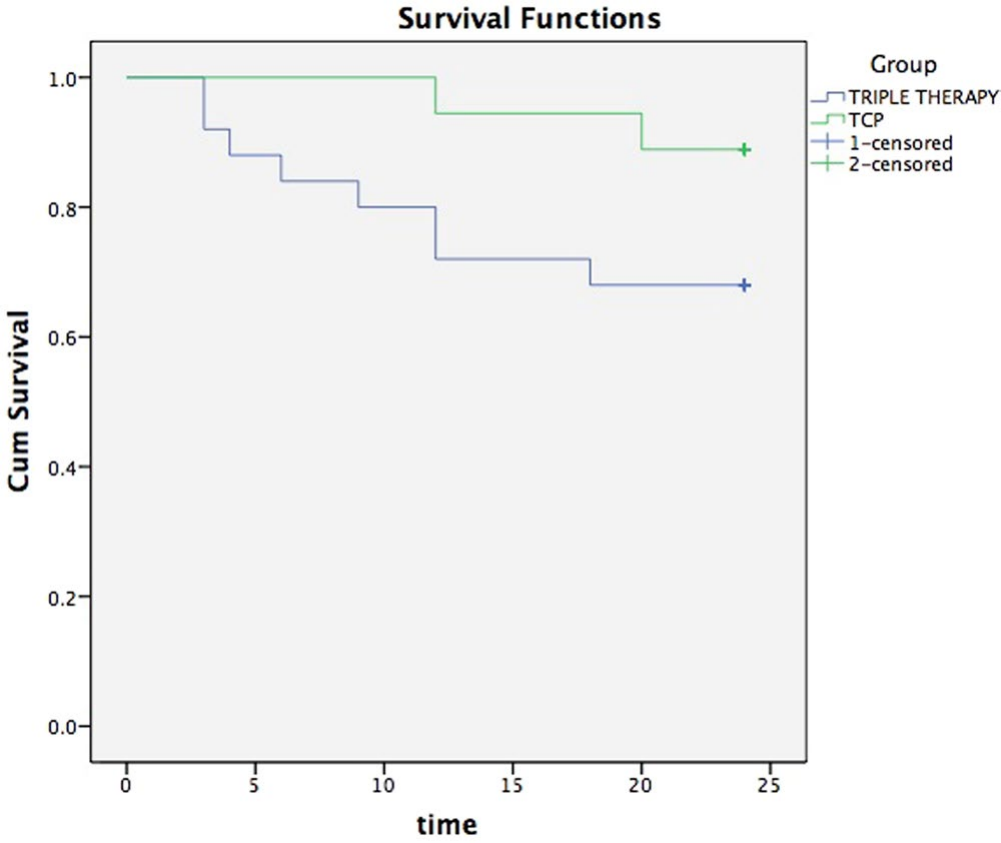
The cumulative survival curve.

**Figure 5:**
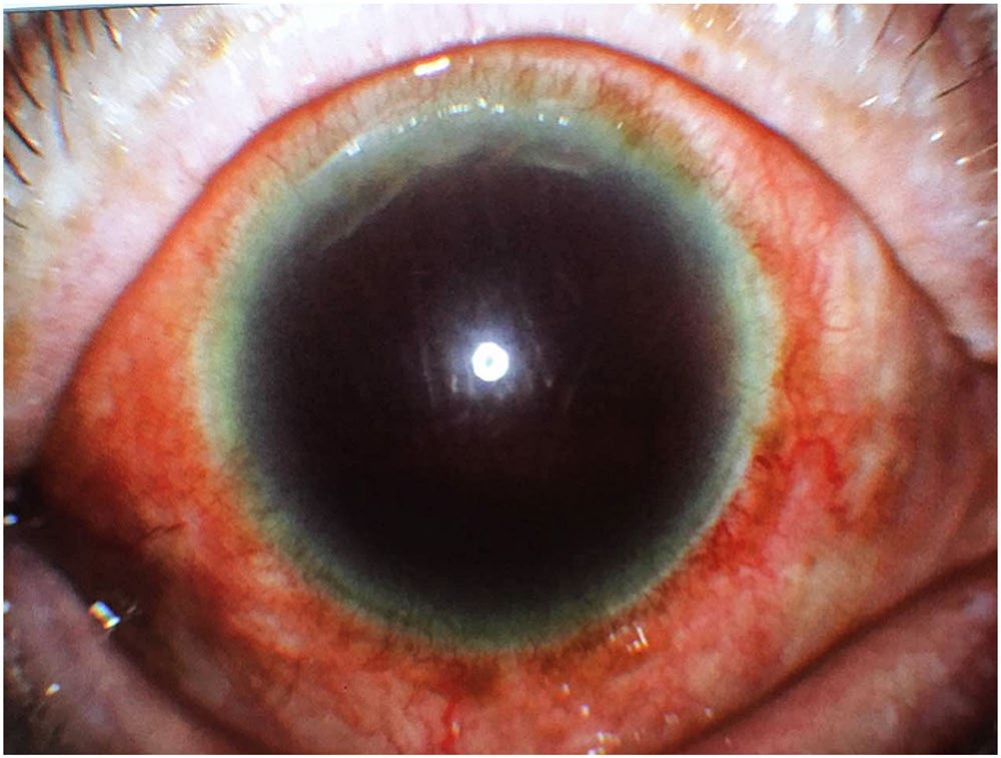
Hypotony with spontaneous bleeding in the anterior chamber after TCP. The eyeball is soft, and corneal folds could be seen. Furthermore, spontaneous bleeding almost filled the anterior chamber. The fundus could not be seen.

At the final follow-up, the relative success rates in the triple sequential therapy group and the TCP group were 89% and 60%, respectively; the difference was significant (χ^2^ = 4.623; P = 0.032; Fig. 4). Patients in the triple sequential therapy group showed no hypotony or eyeball atrophy. There were three cases of hypotony and two cases of eyeball atrophy in the TCP group (Fig. 5). Complications in the triple sequential therapy group included one case of conjunctival incision dehiscence after trabeculectomy with a good prognosis after repeated conjunctival suture, and two cases of recurrence of iris neovascularisation, which regressed due to additional laser photocoagulation. The IOP decreased to the normal level, and there was no recurrence until the final follow-up. Five patients in the TCP group underwent repeated cyclophotocoagulation (twice in three cases and three times in two cases). The average treatment times of TCP was 1.3 ± 0.6.

## Discussion

The current study compared the efficacy between the triple sequential therapy and TCP for treating NVG in the angle-closure stage. The results showed that the IOP of triple sequential therapy group was slightly lower than that of TCP patients. At 36 months after surgery, the success rate of the triple sequential therapy group was significantly higher than that of the TCP group. The rates of preservation of visual acuity and regression of iris neovascularisation in the triple sequential therapy group were higher than those in the TCP group. The incidences of hypotony and eyeball atrophy were 0 in the triple sequential therapy group, but five cases occurred in the TCP group. These results indicate that, compared with TCP, triple sequential therapy is more effective for the regression of neovascularisation and treatment of primary retinal disease, as evidenced by the higher rate of preservation of visual acuity, satisfactory control of IOP, and lower incidence of post-surgery complications.

NVG is a type of refractory glaucoma, which is associated with retinal ischemic diseases in up to 97% of the cases^11^. Ocular ischemic syndrome, DR, CRVO, carotid artery obstruction, and CRAO are the most common causes of retinal ischemic diseases^11^. Clinically, NVG is divided into three stages: the rubeosis iridis, open angle stage, and angle closure stage. The angle closure stage is the terminal stage of NVG, in which the fibrous vascular contraction in the angles of the chamber, peripheral anterior synechiae (PAS), and interconnection cause angle closure and permanent damage to the trabecular meshwork. At this stage, IOP often increases sharply and is difficult to control^12^. Furthermore, patients at this stage experience severe visual impairment. Enucleation, ciliary process destruction, or TCP^13^ may be performed in such patients. However, ciliary process destruction (e.g., ciliary body photocoagulation) does not treat the primary retinal disease. Thus, it is associated with a high probability of neovascular recurrence^6–10,14,15^ and is not conducive to improvements in vision. Furthermore, as it is a non-quantitative treatment and damages the function of the ciliary body, postoperative eyeball atrophy may occur^8,9^. The treatment of neovascular diseases has greatly improved recently due to the application of anti-VEGF in eye diseases^1^. The primary purpose of the triple sequential therapy is to relieve retinal ischemia and preserve the existing visual acuity. Completion of PRP at a minimum cost lies at the core of the treatment. Since anti-VEGF causes regression of new blood vessels, filtration surgery maintains continuous control of IOP, and retinal photocoagulation alleviates the primary disease caused by retinal hypoxia, a combination of the three may help preserve the structural integrity of the eye and partially restore the visual function. The therapy combining intraocular injection of anti-VEGF, trabeculectomy, and PRP via binocular indirect ophthalmoscopy used for NVG in this clinical study requires that the treatments be administered not only in a specific order but also with efficient timing, hence the term triple sequential therapy.

In this study, we compared the triple sequential therapy to TCP, and the results showed that the success rate of the triple sequential therapy (89%) was significantly higher than that of TCP (60%). The extent of the preservation of visual function and regression of iris neovascularisation after triple sequential therapy was significantly higher than that after TCP. There were no significant differences in the reduction of IOP or the numbers of anti-glaucoma drugs used after surgery between the triple sequential therapy and TCP groups. This result may be attributed to the small number of cases included in this study. However, the results indicated that the effect of the triple sequential therapy on the reduction of IOP was reliable. It should be mentioned that among the patients who received triple sequential therapy, none required systemic medications to control IOP, and there were no cases of hypotony or eyeball atrophy following the intervention. These results indicated that triple sequential therapy is a reliable method to control IOP in patients with NVG. Furthermore, this approach causes less damage to the eye tissues, which is due to the improvement in retinal hypoxia by the anti-VEGF treatment and PRP. In addition, triple sequential therapy is associated with decreased injury caused by filtration surgery to control IOP compared to ciliary body photocoagulation.

The complications associated with triple sequential therapy included conjunctival bleb wound dehiscence (n = 1) and recurrence of rubeosis of the iris (n = 2). Conjunctival wound dehiscence might be associated with older age and the use of anti-metabolic medicine, and may also be associated with the anti-VEGF intravitreal injection. The affected patient had a good prognosis after conjunctival suture. The two patients with iris neovascularisation recurrence exhibited increased IOP and poor laser spot formation based on the fundus examination because part of the laser energy was absorbed by the lens opacity. After supplemental photocoagulation, rubeosis of the iris regressed and IOP decreased to a normal level. Up until the last follow-up, there was no rubeosis recurrence. Therefore, complete PRP is essential to prevent the recurrence of rubeosis of the iris. For eyes with unclear refracting media, more laser energy can be absorbed by turbid crystalline tissue or the vitreous body, resulting in a reduced laser effect. In such cases, the laser energy should be intentionally increased, patients should be subsequently followed up over time, and photocoagulation should be supplemented. Although cataract surgery can improve the translucency of the refracting media, the risk of surgery is high in such patients, the postoperative inflammatory response is strong, and the benefit of controlling fundus diseases remains to be determined. The rate of regression of iris vascularisation was 40% in the TCP group, which is consistent with 36% in a previous report^8^. The incidence of eyeball atrophy and hypotony was 20%, also in line with the 27% of the previous study^8^.

Although no comparison between the efficacy of TCP and triple sequential therapy for treating NVG have been reported, some studies have analysed the success rate of trabeculectomy after anti-VEGF therapy. Higashida *et al*.^2^ reported a 1-year success rate of 87% in patients with NVG following trabeculectomy after anti-VEGF injection and who had received retinal laser therapy, which is consistent with our present findings. Another study by Kobayashi *et al*.^3^ also reported the 1-year success rate of trabeculectomy after anti-VEGF injection to be at 83.3%. The slightly higher success rate of the surgery in our study may be associated with the subsequent use of PRP.

The current common treatments for NVG also include drainage valve implantation. Although anti-VEGF intravitreal injections can greatly reduce intraoperative bleeding, the 6-month success rate of the surgery is only 71–73%^4,5^, while the 1-year success rate is 63–72%^4,16^. All these reported rates are significantly lower than the rate observed in the present study, which may be associated with the lack of subsequent pan-retinal laser treatment. A previous study has recruited patients with NVG whose IOP was not well controlled even after retinal photocoagulation^17^. After receiving anti-VEGF treatment followed by trabeculectomy or drainage valve implantation, the success rates 6 months after surgery were 94% in the trabeculectomy group and 68% in the valve implantation group. The author speculated that the cause of this difference might be associated with the greater trauma following drainage valve implantation and the early high incidence of postoperative complications, such as shallow anterior chamber and anterior chamber haemorrhage^17^. Indeed, anti-neovascularisation therapy is crucial for NVG^18^.

Compared with previous studies, the PRP in our present study was performed using indirect ophthalmoscopy laser photocoagulation technology. The procedure had no direct contact with the eyeball and avoided the risk of conjunctival wound dehiscence, infection, and compression of filtering blebs that could be caused by the pressure exerted on the eyeball surface. This is in contrast to the direct PRP, which is performed via Goldmann three mirror lenses after filtering surgery. The triple sequential therapy reduced the interval between the filtering surgery and the subsequent PRP, which was beneficial for the completion of the pan-retinal photocoagulation within the effective period of anti-VEGF therapy. The triple sequential therapy is advantageous not only to control effectively retinal hypoxia, prevent the recurrence of iris neovascularisation, and reduce complications but also to help preserve the filtering function after anti-glaucoma filtering surgery, which effectively controls IOP.

The limitations of this study were the small number of cases and the limited follow-up time.

In summary, triple sequential therapy is a safe and reliable treatment for NVG at the angle closure stage. Moreover, PRP could be completed within the period of effective anti-VEGF therapy, and the drainage effect of filtering surgery prevented the recurrence of iris neovascularisation. Compared with TCP, triple sequential therapy had a higher preservation rate of the visual function, a satisfactory rate of IOP control, and a lower incidence of associated complications. These features are advantageous for the preservation of the visual function and controlling IOP.

## Methods

Eighteen patients (18 eyes) diagnosed with NVG in the Department of Ophthalmology in Peking University Third Hospital from May 2014 to May 2016 were analysed retrospectively. Written informed consent was obtained from all participants, and the study was carried out with the approval of the Institutional Reviewer Board of Peking University Third Hospital(No. 2014-0037). The study adhered to the tenets of the most recent revision of the Declaration of Helsinki. The general information, BCVA, IOP, and the number of anti-glaucoma drugs were recorded before and after therapy. The anti-glaucoma drugs in this study included systemic drugs (intravenous infusion of 20% mannitol, oral Nimox, or isosorbide) and topical drugs (beta-blockers, alpha agonists, carbonic anhydrase inhibitors, and prostaglandin analogues). The results of the silt lamp and fundus were recorded before and after treatment.

The following inclusion criteria were used: (1) patients diagnosed with NVG combined with retinal lesions; (2) patients who received the maximum dose of anti-glaucoma drugs, with IOP > 21 mmHg; (3) no significant vitreous haemorrhage or hyphema in the anterior chamber; and (4) patients who could tolerate ophthalmic surgeries. Excluded from the study were patients (1) with congenital anomalies of the eye; (2) obvious inflammation of the eye; (3) who had received anti-glaucoma surgeries; (4) with retinal detachment; or (5) with severe systemic diseases.

### Treatment

#### Triple sequential therapy

Anti-VEGF intravitreal injection: All patients were in a supine position during the procedure. After the IOP was reduced by anterior chamber puncture, the needle was inserted from the flat area of the ciliary body located 3.5 mm posterior to the corneal limbus, and 0.5 mg (0.05 mL) ranibizumab (Lucentis; Novartis Pharmaceuticals, Basel, Switzerland) was injected into the vitreous body^19^. The IOP was measured 2 hours after the intravitreal injection. If the IOP was >40 mmHg and the patient presented with distending pain in the eyes and a headache, IOP-reducing drugs were administered. If necessary, an anterior chamber puncture was performed.

Trabeculectomy: Trabeculectomy was performed 3 to 5 days after the anti-VEGF intravitreal injection. A fornix-based conjunctival flap and a 4 × 3 × 3 mm trapezoidal scleral flap were created. Subsequently, 0.2 g/L mitomycin (10 mg/10 mL; Zhejiang Hisun Pharmaceutical Co., Zhejiang Province, China) was placed under the conjunctiva and the scleral flap. The cotton pad was removed after 3 min, and 150 mL saline were used for irrigation. Approximately 1 mm × 3 mm of trabecular meshwork was resected, and the scleral flap and conjunctival tissue were sutured. Routine anti-infection and anti-inflammatory treatments were performed postsurgery.

Pan-retinal photocoagulation (PRP) via indirect ophthalmoscopy: Due to the unclear refracting media and non-ideal dilation of pupils, the conventional PRP with slit lamp delivery could not be performed. Therefore, 5–7 d after trabeculectomy, PRP was initiated via binocular indirect ophthalmology. The laser spot diameter was 200–300 μm, the energy was 250–400 mW, and a total of 1,000–2,000 micro-burns were made. Each eye received at least two photocoagulation treatments with a 1-week interval. The triple sequential therapy was completed within 1 month.

#### Transscleral cyclophotocoagulation (TCP)

The TCP was performed after injecting 5 mL of 2% lidocaine to induce retrobulbar anaesthesia. An OcuLight SLx 810 nm diode laser photocoagulator and laser guiding fibres (IRIS Medical Instruments, Flevoland, Netherlands) were used. The initial laser energy setting was 1,800 mW and the treatment duration was 2,000 ms. If a ‘pop’ sound could be heard during laser delivery, the energy setting was maintained; if the ‘pop’ sound was inaudible, the energy setting was increased. During laser delivery, the probe was placed 1.5 mm posterior to the corneal limbus. The area covering 3/4 of the circumference of the ciliary body and generally 20 to 22 spots were selected, and the anterior ciliary arteries at the 3 o’clock and 9 o’clock positions were avoided. Patients received routine anti-inflammatory and anti-infective treatments after surgery. For repeated treatment, 90 degrees of the ciliary body were reserved from photocoagulation^20^.

### Evaluation of the therapeutic effect and follow-up

The BCVA, IOP, use of anti-glaucoma drugs, neovascular regression, and fundus lesions were observed after treatment. The criteria of absolute success were an IOP of 6 to 21 mmHg and no need for anti-glaucoma drugs. The criteria of relative success were an IOP of 6 to 21 mmHg and the use of two or more types of anti-glaucoma drugs^21,22^. The BCVA, which was compared before and after treatment, and the results of the final follow-up were used as the evaluation indexes. The follow-up period was 12 to 36 months, with an average of 20.1 months; each patient was followed up for no less than 12 months.

### Statistical analysis

All data were analysed by SPSS Statistics for Windows Version 17.0 software (SPSS, Inc., Chicago, IL, USA). The BCVA before and after surgery, IOP, and number of anti-glaucoma drug types were presented as mean ± standard deviation. The least-significant difference test was used for intra-group comparison, and the chi-square test was used to compare the number of patients between groups who exhibited preserved or improved vision after different treatments. Statistical significance was considered at *P* < 0.05. A Kaplan-Meier estimate was used to calculate the survival curves (relative success rate).
